# Editorial: Second language learning and neuroplasticity: individual differences

**DOI:** 10.3389/fpsyg.2024.1417238

**Published:** 2024-05-15

**Authors:** Jing Yang, Fan Cao, Walter J. B. van Heuven, Leilei Mei

**Affiliations:** ^1^School of International Studies, Zhejiang University, Hangzhou, China; ^2^Department of Psychology, The University of Hong Kong, Pok Fu Lam, Hong Kong, Hong Kong SAR, China; ^3^School of Psychology, University of Nottingham, Nottingham, United Kingdom; ^4^Philosophy and Social Science Laboratory of Reading and Development in Children and Adolescents (South China Normal University), Ministry of Education, Guangzhou, China

**Keywords:** second language learning, brain plasticity, magnetic resonance imaging, individual differences, interpersonal neural synchronization

The past decades witnessed enormous growth of interests in brain mechanisms underlying second language (L2) learning. Using a variety of neuroscience methods, such as functional magnetic resonance imaging (fMRI) and event-related potentials (ERPs), researchers have investigated functional and structural brain changes induced by L2 learning (e.g., Wong et al., 2007; Li et al., 2014; Pliatsikas et al., 2015; for a review, van Hell, 2023). Key modulators, such as L2 age of acquisition (e.g., Perani et al., 1998; Hernandez and Li, 2007), L2 proficiency (DeLuca et al., 2019; Wang et al., 2020), L2 immersion experience (e.g., Stein et al., 2014), and language learning duration (Berken et al., 2016), have also been investigated. Nevertheless, our understanding of L2-learning-related neuroplasticity and individual differences is still in its infancy compared to L1 acquisition. This Research Topic calls for neurolinguistic researchers to present their latest findings and perspectives on L2 learning and individual differences.

Our call resulted in 5 seminal works (4 original research papers and one review paper) that used different perspectives. Specifically, Tu et al. tapped into the brain anatomical changes induced by learning Chinese as a second language using structural MRI, while Dong et al., in an fMRI study, examined adults' preference for the phonology pathway when learning a new phonology. To reveal the influence of context on L2 learning and processing, Zhuang et al. showed that changing facial contexts influences bilinguals' language production via their reactive control mechanism in an ERP study, which provides empirical evidence for the adaptive control model (Green and Abutalebi, 2013). Unlike most research on L1 and L2 learning, Zhang et al. focused on the interpersonal neural synchronization (INS) during teaching-learning context collected using functional near-infrared spectroscopy (fNIRS). They conducted a meta-analysis of 16 published papers, which revealed that the INS is a significant predictor of L2 learning outcomes. The final contribution to our Research Topic is a review of brain research on Chinese reading development and reading disability by Cao, in which she summarized the language-general and language-specific neural mechanisms of Chinese reading, reading development, and dyslexia. The neural mechanisms of L2 learning was not addressed in this review and therefore would be interesting for future work.

## Individual differences in l2-learning-induced brain anatomical changes

Does language learning induce brain anatomical changes in adults? There has been an increasing interest in neuroplasticity and L2 learning. However, the majority of the research has focused on learning alphabetic languages. To explore Chinese-learning-induced brain neuroplasticity in adults, Tu et al. compared 17 Indian students in China who learned Chinese for more than three years with 21 Indian students who also live in China but who did not have knowledge of Chinese. Both groups made judgements whether the presented Chinese characters contained specific Chinese radicals. MRI data analysis showed that Chinese L2 learners had greater gray matter volume (GMV) in the lingual gyrus than the controls, and the learners' L2 learning duration positively correlated with their GMV in the left inferior frontal gyrus. Furthermore, both groups' behavioral accuracy in the Chinese character recognition task significantly and positively correlated with their GMV in the left lingual gyrus and fusiform gyrus. The authors suggested that learning Chinese as a second language induced neuroplasticity in L1 and L2 brain areas, which was modulated by language-specific features.

## Individual preference on reading pathway in l2 phonology learning

Neuroimaging studies have revealed two pathways involved in word reading: the lexical pathway for reading irregular words and the sublexical pathway for reading words following grapheme-to-phoneme rules. Dong et al. examined individual preferences for using those two pathways when learning 16 artificial characters through address-phonology (whole-word to whole-word phonology mapping) and another 16 characters via assembled-phonology (grapheme-to-phoneme mapping) in an fMRI study. Interestingly, they found that participants' preference for the lexical pathway was associated with their superior performance in addressed phonology learning. Moreover, the preferred lexical pathway during novel phonology learning involved less neural activation.

## Individual adaptation in bilingual production to changing contexts

Context is essential for language learning and processing. Studies have shown that facial cues, such as faces with ethical features (e.g., Caucasian, Asian), influence bilinguals' language production and comprehension (Li et al., 2013; Yang et al., 2018; Peeters, 2020). Zhuang et al. examined bilinguals' language control changes when the interlocutor's face-language matching varied. In this ERP study, non-proficient Chinese-English bilinguals performed mixed-language picture naming tasks in three sessions with varying face-language congruency: 25%, 50%, and 75%. Their results revealed that bilinguals' reactive control, instead of proactive control, is modulated by the changing facial contexts, highlighting the vital role of context in modulating language processing and learning.

## Interpersonal neural synchronization: a new biomarker for learning outcomes

Individual differences in L2 learning are commonly indicated by distinct performance and neural activity patterns. Researchers increasingly use fNIRS to track interpersonal neural synchronization (INS) during teaching-learning interaction. In a meta-analysis, Zhang et al. examined the role of INS in predicting learning outcomes. They evaluated 16 studies with different samples and examined modulation effects of the style, mode, content, as well as the assessment method of learning outcomes. The INS revealed a positive correlation with learning outcomes, which was influenced by interaction style and mode. This meta-analysis took an interactive perspective on individual differences in L2 learning and suggested that the INS could be a valid biomarker for L2 learning achievement.

## Language differences and language learning difficulties: an overview of Chinese dyslexia research

This Research Topic also attracted researchers who explore the influence of language features on language learning difficulties. Specifically, Cao reviewed neuroimaging studies investigating Chinese reading development and reading disability that focused on developmental changes in neural functional differences in Chinese dyslexic children and adults. Studies on Chinese reading showed that Chinese reading shifts from an early reliance on phonology for meaning in children to direct orthography-to-meaning mapping in adults. Chinese reading development, therefore, is characterized by decreasing involvement of the phonology pathway and increasing dependence on the orthographic pathway. Further, unlike alphabetic languages, Chinese is a morpho-syllabic language with complex visual configuration of the writing units (characters). Reading development in Chinese strongly correlates with learners' morphological awareness and visual attention. Chinese dyslexics showed delayed development of the phonological reading, characterized by decreased activation in the left IFG and disability in shifting to the left inferior temporal gyrus, responsible for orthographic processing.

In sum, this Research Topic presents readers with multidisciplinary approaches to understanding L2-learning-related neuroplasticity and individual differences. It showed new directions for research on L2 learning in the brain, suggests the influence of context on L2 learning and processing, provides new biomarkers for predicting L2 learning performance, and sheds light on language-specific brain mechanisms for language learning and disability. We hope this Research Topic inspires future research into the neurocognitive mechanism of L2 learning.

## Author contributions

JY: Conceptualization, Funding acquisition, Investigation, Project administration, Resources, Validation, Writing – original draft, Writing – review & editing. FC: Conceptualization, Investigation, Methodology, Project administration, Resources, Supervision, Writing – review & editing. WH: Conceptualization, Investigation, Methodology, Project administration, Resources, Supervision, Writing – review & editing. LM: Conceptualization, Investigation, Methodology, Project administration, Resources, Supervision, Writing – review & editing.
